# Adversarial Networks for Scale Feature-Attention Spectral Image Reconstruction from a Single RGB

**DOI:** 10.3390/s20082426

**Published:** 2020-04-24

**Authors:** Pengfei Liu, Huaici Zhao

**Affiliations:** 1 Shenyang Institute of Automation, Chinese Academy of Sciences, Shenyang 110016, China; hczhao@sia.cn; 2 Institutes for Robotics and Intelligent Manufacturing, Chinese Academy of Sciences, Shenyang 110016, China; 3 University of Chinese Academy of Sciences, Beijing 100049, China; 4 Key Laboratory of Opto-Electronic Information Processing, Chinese Academy of Sciences, Shenyang 110016, China; 5 The Key Lab of Image Understanding and Computer Vision, Shenyang 110016, China

**Keywords:** hyperspectral imaging, generative adversarial network, attention mechanism, feature pyramid, boundary supervision

## Abstract

Hyperspectral images reconstruction focuses on recovering the spectral information from a single RGBimage. In this paper, we propose two advanced Generative Adversarial Networks (GAN) for the heavily underconstrained inverse problem. We first propose scale attention pyramid UNet (SAPUNet), which uses U-Net with dilated convolution to extract features. We establish the feature pyramid inside the network and use the attention mechanism for feature selection. The superior performance of this model is due to the modern architecture and capturing of spatial semantics. To provide a more accurate solution, we propose another distinct architecture, named W-Net, that builds one more branch compared to U-Net to conduct boundary supervision. SAPUNet and scale attention pyramid WNet (SAPWNet) provide improvements on the Interdisciplinary Computational Vision Lab at Ben Gurion University (ICVL) datasetby 42% and 46.6%, and 45% and 50% in terms of root mean square error (RMSE) and relative RMSE, respectively. The experimental results demonstrate that our proposed models are more accurate than the state-of-the-art hyperspectral recovery methods

## 1. Introduction

Hyperspectral imagery can provide richer information than ordinary cameras, and can be used for a variety of applications, such as image classification [1], understanding environmental changes, and so on. Traditional cameras use sensor filters to transform the incoming light spectra into three color channels, but the projection process leads to the loss of multiple spectral signals. The missing spectral data play an important role in classifying objects based on their spectral properties. Thus, hyperspectral imaging has become an active area of research [2,3,4,5,6]. Reconstructing hyperspectral image for every pixel is a severely ill-posed problem.

To obtain narrowband hyperspectral data in consecutive wavelengths, a number of hardware design methods have been proposed [7,8,9]. The approaches involve carefully designing the lighting sources, reducing the resolution in one of the acquisition axes (spatial, temporal), and using multiple color filters to complete the reconstruction [10,11,12]. However, these methods rely on rigorous environment conditions and extra equipments.

Recently, hyperspectral reconstruction from a single RGB image has attracted considerable attention due to its rapid speed, easy-access, and low cost. The core of this method involves exploiting the correlation between RGB values and their corresponding hyperspectral radiance [3,13]. Arad et al. [3] created a sparse dictionary of hyperspectral signatures and their corresponding RGB projections, which could then be used as a priori information to map RGB vectors to spectra. These solutions often learn non-linear mapping from RGB to hyperspectral images based on a large number of training data.

Convolutional neural networks (CNNs) have achieved success in various computer vision tasks. CNN-based methods were also introduced for hyperspectral recovery [4,14,15,16]. Nguyen et al. [14] used a radial basis function (RBF) network to learn the mapping from RGB to spectra. More recently, Yan et al. [16] applied the traditional isometric feature mapping (Isomap) algorithm to reduce the dimensions of hyperspectral data, then trained a neural network based on nonlinear mapping between the RGB color space and three-dimensional embedding. Xiong et al. [4] developed a unified deep learning framework, named hyperspectral convolutional neural network (HSCNN), for hyperspectral recovery from both RGB and compressive measurements. These methods were proven to obtain state-of-the-art results on the ICVL dataset [3]. However, the manifold reduction method requires knowing the spectral response of the RGB camera in advance, and the upsampling process in HSCNN also requires the prior understanding of an explicit spectral response function corresponding to the integration of hyperspectral radiance to RGB values. This restricts the applicability of this kind of method when the spectral response function is unknown or difficult to obtain in practice.

Generative adversarial networks (GANs) have been vigorously studied and have been proven to be suitable for image-to-image translation tasks. The discriminator improves the image quality when the fake image is blurred from the generative model. GANs can learn the mapping, adapting to the training data with different kinds of loss functions. We propose a low cost and learning-based end-to-end approach to reconstruct the spectra from a single RGB image. The pixel-to-pixel GAN [17] offers a GAN-based framework for a variety of applications. We used this GAN-based algorithm to learn a generative model of the joint spectro-spatial distribution of the data manifold of natural hyperspectral images. We selected the generator U-Net, which provides the best performance amongst the available alternatives. However, U-Net has the disadvantage of low quality and low resolution, blurring the detail when zooming out the images. To solve this problem, we replaced U-Net with our proposed scale attention pyramid network, named SAPUNet, which overcomes the problem. Based on SAPUNet’s promising results, we propose another distinct architecture that replaces the U-Net structure with W-Net with boundary supervision.

Our contributions can be summarized as follows:

(1) We present a novel end-to-end GAN-based approach for hyperspectral reconstruction that requires only a single RGB image. The proposed pipeline reconstructs the hyperspectral data without requiring of the spectral response function in advance.
(2) We propose SAPUNet, which optimizes the U-Net architecture by using scale attention modules to fuse local and global information. The feature pyramid and attention mechanism inside the network for feature selection improves the accuracy of hyperspectral reconstruction.
(3) We further designed the W-Net structure based on SAPUNet using boundary attention with a feature fusion scheme, deriving SAPWNet, which performed the best on the ICVL dataset.

## 2. Related Work

A number of methods have been proposed to address this hyperspectral recovery task. Some snapshot hyperspectral cameras are designed for obtaining spectral signals [2,18]. A combination between hyperspectral and RGB cameras was developed for capturing hyperspectral data at high spatial and spectral resolution [9,13,19]. Oh et al. [12] reconstructed hyperspectral images using multiple consumer digital cameras, using different RGB cameras with different spectral sensitivities. Takatani et al. [11] proposed a low-cost algorithm by augmenting a consumer camera with a tube of reflectors, but this method to sacrifices the spatial or spectral resolution. Kawakami et al. [20] proposed a method that fuses a low-resolution hyperspectral image with a high-resolution RGB image to obtain a high-resolution hyperspectral image. These methods always require many components or rely on rigorous environment conditions. Obtaining hyperspectral images via a single RGB image would be convenient to implement, portable, and affordable.

The more recent hyperspectral reconstruction algorithms using only a single RGB image without any extra equipment are more effective. Arad et al. [3] collected prior hyperspectral data for the construction of a sparse hyperspectral dictionary based on a sparse dictionary. However, the method is dependent on the dictionary. Akhtar et al. [13] developed data clustering with a Gaussian process to replace the dictionary atoms. Aeschbacher et al. [21] developed A+ [22] from super-resolution to hyperspectral reconstruction. Antonio et al. [23] proposed using a constraint sparse coding method to reconstruct illumination-free spectra based on learning the prototype set. Yan et al. [16] used isometric feature mapping to reduce natural scene spectra to a low dimensional space, then transformed three-to-many mapping (RGB to spectrum) to three-to-three mapping (RGB to 3D embedding of spectra), finally using a low-dimensional manifold reconstruction method for spectral recovery. This approach avoided establishing any priors based on the reduction from three-to-many to three-to-three mapping. This is typically accomplished by knowing the spectral response of the consumer RGB camera in advance.

CNNs have now become the dominant approach in different vision challenges due to the ability to automatically extract useful features. More efficient and powerful frameworks are more generalized, such as AlexNet [24], ResNet [25], and DenseNet [26]. They use 1×1 convolution instead of the fully connected layer to generate the heatmap and some deconvolution layers are used for pixel-wise labelling. Qiu et al. [27] used CNN to analyze spectral data to identify rice seed varieties. Nguyen et al. [14] proposed learning mapping from white-balanced RGB values to reflectance spectra based on a radial basis function (RBF) network. Xiong et al. [4] proposed a CNN structure suitable for this task from super-resolution and obtained promising results. Based on [4], Shi et al. [28] replaced plain convolution with residual block, adopted the dense structure to replace residual block, and produced more accurate results. Gallinani et al. [29] used a CNN-based encoder-decoder structure to recover hyperspectral signals. Alvarez-Gila et al. [30] proposed spatial context-aware adversarial UNet-GAN (SCAUNet-GAN) for hyperspectral reconstruction, which uses U-Net [31] as the generator.

Compared with the above methods, our proposed models offer more accurate solutions for improving the image detail of hyperspectral reconstruction. We developed a U-Net structure with scale attention pyramid modules, which are directed to form a feature pyramid at each level. We proposed W-Net, which has dilated convolution that produces even more accurate results.

## 3. Adversarial Spectra Reconstruction via RGB

This section describes the core model of our methods, along with some of the important GAN development in our proposed models.

### 3.1. Analyzing the Physical Model of Natural Spectra Reflectance

We wanted to reconstruct the hyperspectral information from a single RGB image of a natural scene. This appears to be a server ill problem, that involves three-to-many mapping. The spectra of natural scenes lie in a low-dimensional manifold [16]. The mathematic model can be expressed by

(1) $$i\left(\lambda \right)=l\left(\lambda \right)r\left(\lambda \right)$$

where $l\left(\lambda \right)$ and $r\left(\lambda \right)$ represent the illumination and reflectance intensity at wavelength λ. If we stack all spectra into a matrix *I*, we obtain

(2) $$I=\left[\begin{array}{ccc} i_{1}\left(\lambda_{1}\right) & \cdots & i_{M}\left(\lambda_{1}\right)\\ \cdots & \cdots & \cdots \\ i_{1}\left(\lambda_{N}\right) & \cdots & i_{M}\left(\lambda_{N}\right) \end{array}\right]=\left[\begin{array}{ccc} l\left(\lambda_{1}\right) & 0 & 0\\ 0 & \cdots & 0\\ 0 & \cdots & l\left(\lambda_{N}\right) \end{array}\right]\left[\begin{array}{ccc} r_{1}\left(\lambda_{1}\right) & \cdots & r_{M}\left(\lambda_{1}\right)\\ \cdots & \cdots & \cdots \\ r_{1}\left(\lambda_{N}\right) & \cdots & r_{M}\left(\lambda_{N}\right) \end{array}\right]$$

where *M* and *N* denote the number of pixels and the number of bands, respectively. The rank of *I* is low-dimensional; thus, three-to-many mapping is achievable due to the sparsity of the natural hyperspectral information, and the response of the RGB sensor may reveal more of the spectral signature [3].

### 3.2. Adversarial Learning

Compared with CNNs, training GANs does not require any approximation method. GANs have attracted attention due to their demonstrated ability to generate real samples following the underlying data densities [32]. The discriminator *D* aims to distinguish between fake data, which are synthesized by generator *G* and real training data, whereas *G* learns to fool the discriminator by generating samples as close as possible to the probability distribution of real images. GANs are generative models that learn mapping from a random noise vector *z* to output image *y*, whereas conditional GANs learn mapping from an observed image *x* and random noise vector *z*. Such conditioning input has been proved useful for more sophisticated applications [17].

The classic GAN architecture is instable. The gradient vanishing problem is caused by the sigmoid cross-entropy loss function; the effective solution involves adopting Earth-Mover (EM) distance or Wasserster-1 as loss function for the discriminator [33,34]. A novel discriminator with an infinite ability to separate real from generated samples was designed for optimizing and computing loss function [34]. GANs with infinite modelling ability are probably the reason for collapsed generators.

Related to conditional GANs, the vanilla GAN objective is always adopted as a loss [35]. However, it suffers from training instability. Wasserstein GAN (WGAN) [15] overcame the problem by using weight clipping to enforce a Lipschitz constraint on the critic. We employed the WGAN objective function as the adversarial loss function. The objective of a conditional WGAN is expressed as:

(3) $$L_{adv}=\min_{G}\max_{D\in R}\underset{I_{rgb},I_{hs}∼p_{data}\left(I_{rgb},I_{hs}\right)}{E}\left[\log D\left(I_{rgb},I_{hs}\right)\right]-\underset{I_{rgb}∼p_{model}\left(I_{rgb}\right)}{E}\left[\log\left(1-D\left(G\left(I_{rgb}\right)\right)\right)\right]$$

where *R* is the set of 1-Lipschitz function, $I_{hs}$ is the original hyperspectral image, $I_{rgb}$ denotes the corresponding RGB image, $p_{data}\left(I_{rgb},I_{hs}\right)$ is the data distribution, and $p_{model}\left(I_{rgb}\right)$ is the model distribution. The RGB image is the only input to *G*. When training, *G* tries to minimize the adversarial loss, while *D* tries to maximize it.

To learn a mapping exactly from a 3-dimensional image to 31-dimensional spectral channels, we used the L1 distance, named content loss, to guide the generator to be close enough to the ground truth. Combining content loss with an adversarial objective can produce more spatially consistent and less blurred results. The final objective including adversarial and content loss then becomes

(4) $$L^{*}\left(G,D\right)=L_{adv}+\lambda L_{1}\left(G\right)=L_{adv}+\lambda \underset{I_{rgb},I_{s}∼p_{data}\left(I_{rgb},I_{hs}\right)}{E}[{∥I_{hs}-G\left(I_{rgb}\right)∥}_{1}]$$

where λ is the scalar weight used to leverage the two loss terms, which was set to 100 in all experiments. Although using L1 loss only fails to reconstruct high-frequency crispness, it can capture the global image structure, for which we do not need an entirely new framework to enforce correctness for global information.

**Figure 1:**
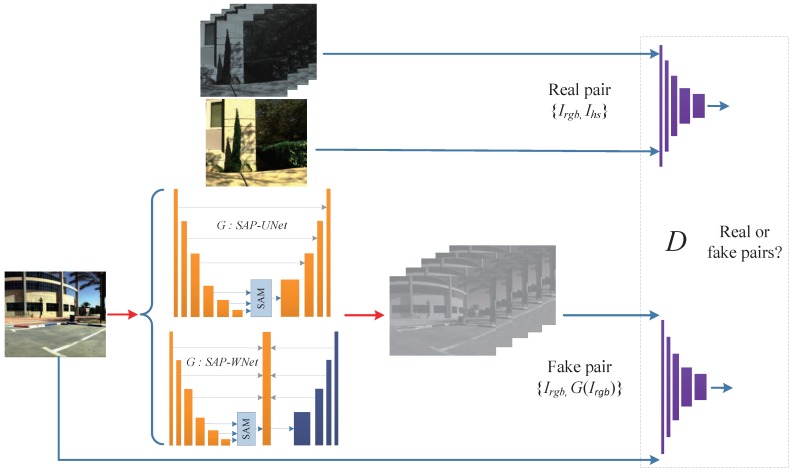
Overview of our two adversarial feature pyramid spectral reconstruction models. SAM, scale attention modules.

The flow of our methods for spectral reconstruction is shown in Figure 1. We first prepared entirely aligned RGB and hyperspectral image pairs from the ICVL dataset, which were extracted as real pairs. Then, we proposed two generator structures, SAPUNet and SAPWNet, which establish feature pyramids and use attention mechanism to select feature layers. The generator *G* takes $I_{rgb}$ as the input and generates the corresponding hyperspectral result. The discriminator *D* is now fed two pairs of images and discriminates if they are real or fake. *G* and *D* are both trained iteratively in an adverse manner.

### 3.3. Generator: SAP-UNet Architecture

The encoding structure always adopts feed-forward networks, which increase training loss due to the decreasing gradient with increasing network depth [25]. Deeper networks can lead to better model expressiveness, which can improve the performance of hyperspectral reconstruction. To solve these problems, we first replaced the feed-forward network by a residual network. Each layer in the residual block performs convolutions with filters to generate a heap of feature maps, then these feature maps are batch normalized and passed through a rectifier non-linearity (ReLU) activation function to produce the input of next convolution. A residual block output $x_{l}$ is defined as:

(5) $$x_{l}=F_{l}\left(x_{l-1}:\omega_{l}\right)+x_{l-1}$$

where $F_{l}\left(x_{l-1}:\omega_{l}\right)$ is defined as a sequence of convolutions followed by ReLU and the batch normalization, and $x_{l}$ is the output of *l*th layer. F only computes the residual and adds $x_{l-1}$ instead of calculating $x_{l}$ directly.

Residual blocks transmit gradients directly to the previous layer, and low-level signals can propagate to any high-level feature through identity mapping [36]. These two superior properties are particularly useful for hyperspectral reconstruction because we want a network deep enough to recover image detail and we need low-level features to restore the whole image structure. We can understand these two characteristics according to the gradient flow in the network. By applying the recursion in Equation (3) several times: $\left(x_{l+1}=x_{l}+F_{l+1}\left(x_{l}:\omega_{l+1}\right)=x_{l-1}+F_{l}\left(x_{l-1}:\omega_{l}\right),etc\right)$, we will have:

(6) $$x_{j}=x_{i}+\sum_{m=i}^{j-1}F_{m+1}\left(x_{m}:\omega_{m+1}\right)$$

where $x_{j}$ and $x_{i}$ represent the output of deep and shallow residual blocks, respectively. Thus, any low-level feature can be passed directly into high-level feature space by adjusting the parameters of the residual block. The gradient can be easily calculated by backward propagation. We express the derivative of the loss from the chain rule of backpropagation:

(7) $$\frac{\partial \phi }{\partial \omega_{i}}=\frac{\partial \phi }{\partial x_{j}}\frac{\partial x_{j}}{\partial x_{i}}\frac{\partial x_{i}}{\partial \omega_{i}}=\left(\frac{\partial \phi }{\partial x_{j}}+\frac{\partial \phi }{\partial x_{j}}\sum_{m=i}^{j-1}\frac{\partial F_{m+1}\left(x_{m}:\omega_{m+1}\right)}{\partial x_{i}}\right)\frac{\partial x_{i}}{\partial \omega_{i}}$$

Gradient propagation is decomposed into two directions, $\frac{\partial \phi }{\partial x_{j}}$ and $\frac{\partial \phi }{\partial x_{j}}\begin{array}{c} \sum_{m=i}^{j-1}\frac{\partial F_{m+1}\left(x_{m}:\omega_{m+1}\right)}{\partial x_{i}} \end{array}$. The former ensures the gradient is transmitted directly to the shallow network; the latter guarantees that the transmission will not disappear. This delivery ensures that deeper network structures can be trained.

We used U-Net based on dilated convolution to extract features. The dilated convolution has a parameter named expansion rate to the convolutional layer, which defines the spacing of the values when the convolution kernel processes the data. This convolution method can discard the pooled layer to output the full-resolution feature map while still obtaining a large receptive field. We used the output of different blocks of U-Net to form the feature pyramid layer after the pooling layer. Thus, the high-level feature layer of the feature pyramid had a larger receptive field, while the lower-level feature layer had a smaller receptive field.

Then we used a scale attention module (SAM), which can produce a scale-level weight matrix by convolution, to indicate which scale should be noticed [37,38]. The scale attention module provides global context prior attention to select the scale-wise feature and fuses the information of three different scale contexts by offering scale-level attention value.

**Figure 2:**
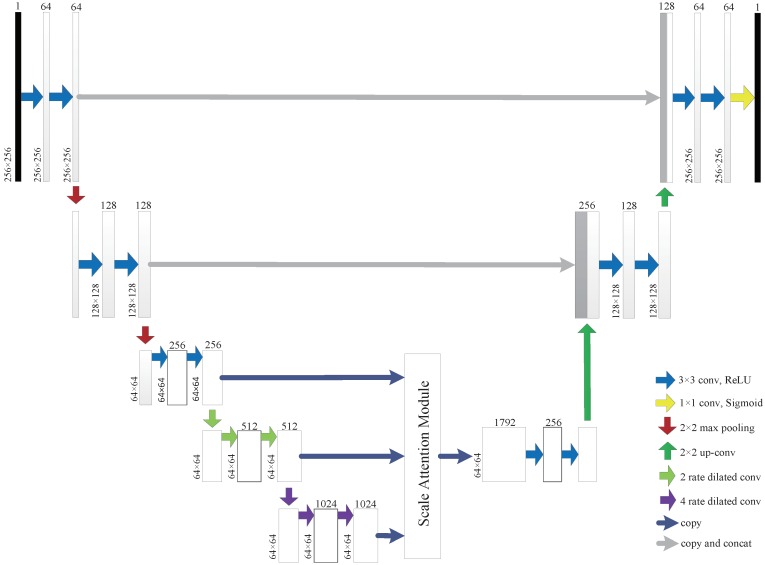
Complete architecture of the Scale Attention Pyramid U-Net with dilated convolution at different rates.

**Figure 3:**
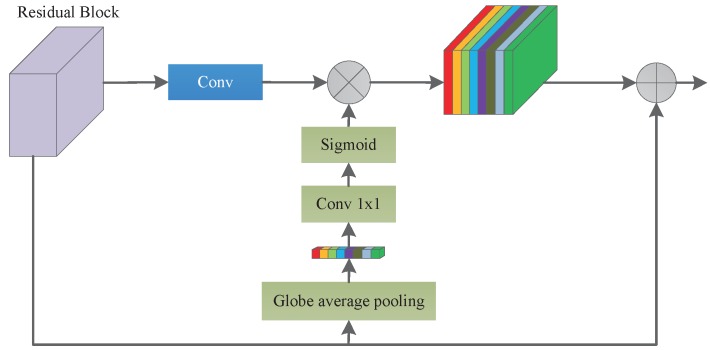
Architecture of the Attention Module Block.

As shown in Figure 2, the SAP-UNet encoding network consists of five large blocks. The size of the feature map for each scale is 1/8 of the input size. To better extract context from different layers, we used the feature map of the last three large blocks after the convolution of different scales to build the feature pyramid. As shown in Figure 3, the bottleneck layer generates an attention feature layer after global average pooling convolution. This global pooling method provides global context as a guidance for feature pyramid to select scale attention. We obtain the attention feature from the global average pooling after 1 × 1 convolution with batch normalization and a sigmoid activation function. Then, we multiplied the attention feature and added the original input to obtain the feature map of the scale layer. Finally, we used bilinear interpolation to adjust a feature pyramid of the same size and performed 3 × 3 convolution to reduce channels after concatenating the feature maps.

### 3.4. Generator: SAP-WNet Architecture

**Figure 4:**
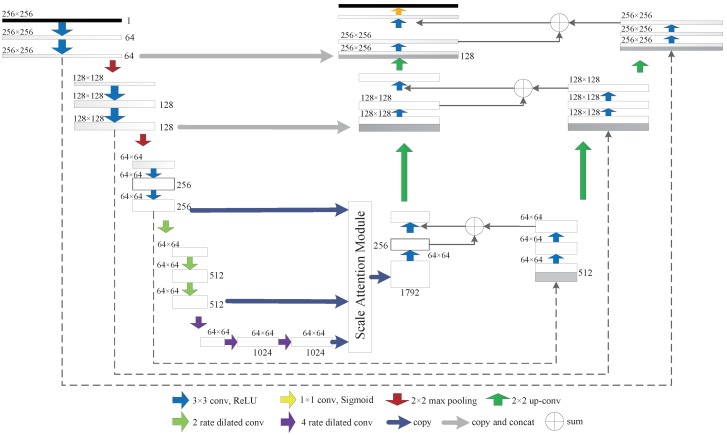
Complete architecture of the Scale Attention Pyramid W-Net with dilated convolution at different rates.

In addition to the U-Net with residual blocks described above, we propose a branch-widening network as an alternative solution, the SAP-WNet model, which is shown in Figure 4. The encoding network is similar to SAP-UNet and employs a scale attention module as well. We widened the structure and imposed a novel branch on the right side to optimize the network training process. We used the edge image extracted by the Canny algorithm to conduct the deep supervision. This method can guide the network to concentrate on reconstructing the recovery image detail through providing more semantic features. W-Net sums the boundary attention feature with the original U-Net on each scale, followed by 3 × 3 convolution and up-convolution to form the final module representation, which contains information about different receptive fields. Finally, the pixel-wise prediction is formed by connecting the fine-grained layers.

The W-Net structure with deep supervision has characteristics suitable for hyperspectral reconstruction. We wanted to reconstruct hyperspectral information from a single RGB image, which is a server ill-posed problem. Insufficient priors would cause edge blurriness in an image that has rich information. However, the pixels with significantly changing intensity values always contain extremely important characters with strong representation. The most important aspect of our design is that we can use boundary supervision to guide the network to identify the edge information with a novel fusion scheme. The concatenation and summation operators on each scale explicitly boosts the feature representation which has the potential to provide a more accurate model. The W-Net model can provide higher reconstruction fidelity, providing sufficient boundary features compared with U-Net.

### 3.5. Markovian Discriminator

In the traditional discriminator structure, a final probability is given with the SoftMax function to process the whole image. However, this does not fit our task, where every image patch must be reserved. We adopted the PatchGAN method [39] that only penalizes structure at the patches scale. A fixed-size patch discriminator can be used for arbitrarily large images. We designed the architecture of PatchGAN as shown in Table 1. This discriminator takes the image as a Markov random field, assuming independence between pixels separated by more than a patch diameter. We used this discriminator convolutionally on the generated image and averaged all responses to offer the ultimate probability.

After the last layer, a convolution was used to map to a 1-dimensional output, followed by a sigmoid active function. Every convolution layer was activated with leaky ReLUs, with a slope of 0.1.

### 3.6. Materials and Implementation Details

Arad et al. [3] created a large database of hyperspectral images of natural scenes. The database images were acquired using a Specim PS Kappa DX4 hyperspectral camera and a rotary stage for spatial scanning. It contains 240 images with 1392 × 1300 spatial resolution over 519 spectral bands (400–1000 nm in roughly 1.25 nm increments). The database includes down-sampled data with 31 spectral channels from 400 to 700 nm in 10 nm increments. It is by far the most comprehensive natural hyperspectral database. We trained our network on 160 pairs of RGB and hyperspectral images from the down-sampled data. We chose 40 images as the validation dataset for parameter fine-tuning. The remaining 40 images were used as the test set to evaluate the performance of the algorithms.

The Chakrabarti dataset consists of 50 images under daylight illumination both outdoors and indoors. The real-world hyperspectral scenes were captured by using Nuance FX, which is a commercial hyperspectral camera. The camera acquires hyperspectral images by sequentially tuning the filter through a series of 31 narrow wavelength bands, approximately 10 nm in bandwidth from 420 to 720 nm.

The network performance was optimized using the Adam solver. We set base learning to 0.0001, reduced by a factor of 0.8 as training error saltation. The momentum and weight decay were set to 0.9 and 0.0001, respectively. The proposed network was trained with an i7-8086K CPU and 2 1080Ti GPUs. Due to the limitation of our computer hardware, we adjusted the original 1392 × 1300 hyperspectral images during the training phase to 256 × 256 images. Training images were resized to 256 × 256 based on the bilinear interpolation algorithm. The generator *G* accepted input images of size 256 × 256 and yielded image sizes of 256 × 256 pixels. Note that we evaluated the final result based on 256 × 256 pixels, not the original size. We set the batch size to 1, which was proven to be effective for image generation tasks during training. We alternated between one gradient descent step on *D*, then one step on *G*. Training was stopped after 700 epochs. Notably, the performance could be improved by increasing the epoch number. For optimization, we trained the models with a combination of adversarial loss and content loss. At inference time, we applied a 50% dropout and instance normalization to obtain the desired results. The training phase required nearly 36 h for the SAPWNet-GAN. All network implementation was based on the top of Pytorch. Pytorch is a fast-maturing deep learning framework being increasingly used by researchers. Pytorch defines mathematical functions and calculates the gradients automatically.

### 3.7. Evaluation Metrics

The performance of the hyperspectral reconstruction was evaluated using four metrics: root mean square error (RMSE), relative RMSE (RMSERel), mean peak signal-to-noise ratio (PSNR) and structural similarity (SSIM).

RMSE was used to evaluate the accuracy of the reconstructed images compared with ground truth. RMSE computes over the spectral dimension for every pixel and averages the entire pixels in the image.

(8) $$\mathrm{RMSE}=\sqrt{\frac{\sum_{i,c}{\left(P_{gt_{i_{c}}}-P_{rec_{i_{c}}}\right)}^{2}}{P_{num}}}$$

where $P_{gt_{i_{c}}}$ and $P_{rec_{i_{c}}}$ denote the value of the *c* spectral channel of the *i*-th pixel in the ground truth and the recovered image, respectively; $P_{num}$ is the size of the hyperspectral image with pixel count multiplies number of spectral channels.

RMSERel represents RMSE relative to the value of the real signal.

(9) $$\mathrm{RMSERel}=\sqrt{\frac{\sum_{i,c}{\left(P_{gt_{i_{c}}}-P_{rec_{i_{c}}}\right)}^{2}}{P_{num}P_{gt_{i_{c}}}}}$$

PSNR is a common metric of the ratio of peak signal and noise.

(10) $$\mathrm{PSNR}=20\cdot \log_{10}\left(\frac{255}{RMSE}\right)$$

SSIM is a classic index of image quality assessment, which is more suitable for human visual perception systems. SSIM evaluates the image quality with the combination of brightness, contrast, and structure.

(11) $$\mathrm{SSIM}\left(p_{rec},p_{gt}\right)=\frac{\left(2\mu_{p_{rec}}\mu_{p_{gt}}+6.5\right)\left(2\sigma_{p_{rec},p_{gt}}+58.5\right)}{\left(\mu_{p_{rec}}^{2}+\mu_{p_{gt}}^{2}+6.5\right)\left(\sigma_{p_{rec}}^{2}+\sigma_{p_{gt}}^{2}+58.5\right)}$$

where $p_{gt}$ is the ground truth, $p_{rec}$ is the recovered image, $\mu_{p_{gt}}$ is the mean of $p_{gt}$, $\mu_{p_{rec}}$ is the mean of $p_{rec}$, $\sigma_{p_{gt}}^{2}$ is the variance of $p_{gt}$, $\sigma_{p_{rec}}^{2}$ is the variance of $p_{rec}$, $\sigma_{p_{rec},p_{gt}}$ is the covariance of $p_{rec}$ and $p_{gt}$.

## 4. Experimental Results and Discussion

This section outlines the quantitative experiments we used to evaluate the performance of our approach on public hyperspectral datasets [3,40]. The two datasets included complex scenarios that cover various materials, shadows, and indoor scenes.

### 4.1. Evaluation on the ICVL Dataset

**Figure 5:**
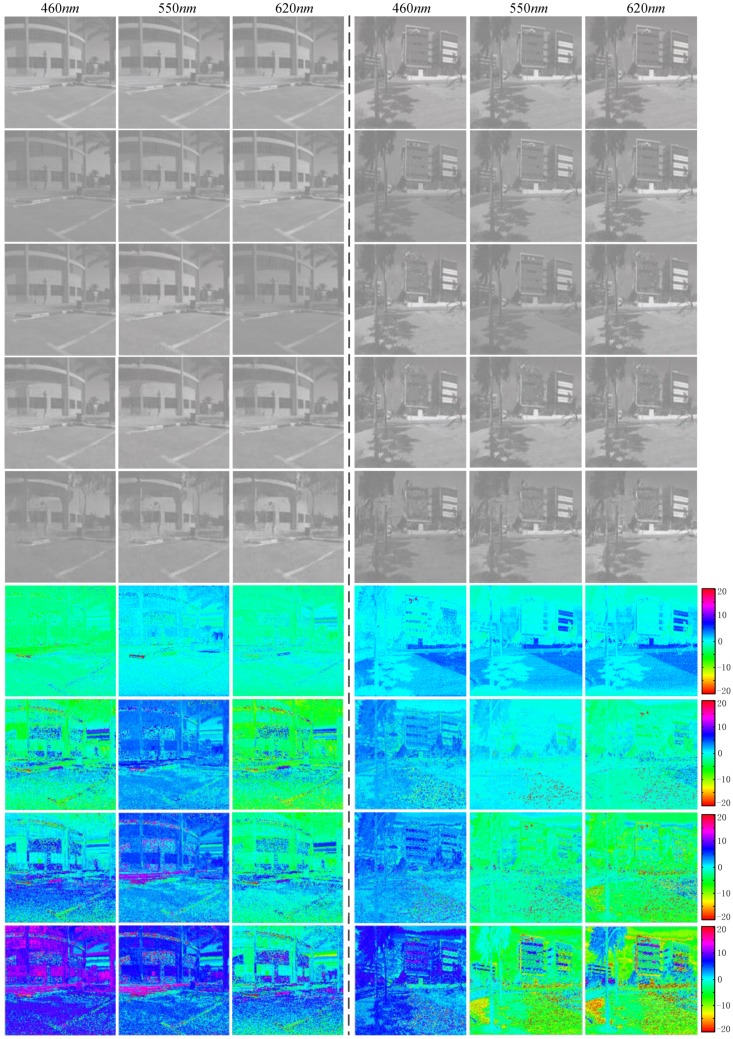
Reconstruction images from the ICVL dataset [3]. From top to bottom, ground-truth, SAPWNet-GAN, SCAUNet-GAN [30], sparse coding [3], and error map of the SAPWNet-GAN.

We evaluate our approach on the ICVL dataset [3], which contains hyperspectral images in natural scenes. Figure 5 depicts the quality of spectra reconstruction obtained with our approach and other algorithms compared to the ground-truth. We selected two images at three different wavelengths to examine the spatial consistency of the results. The error map was calculated using the RMSE on a scale of ±255 [20]. The images recovered using our method were consistently accurate across the wavelength axis irrespective of scene materials. The images recovered by [3,30] contain some artifacts to different degree. The method proposed by Arad et al. [3] is severely dependent on the number of dictionary atoms. A sparse dictionary may produce considerable error, which would contaminate the data. Alvarez-Gila et al. [30] used U-Net to form the SCAUNet-GAN for hyperspectral reconstruction. However, this method only concatenates all the features using the skip-connection, which ignores the semantic gap between different levels. In contrast, we propose a hyperspectral reconstructing learning approach that restores sharp images in an end-to-end manner with no dimension reduction process. We used multi-scale information to synthesize the local and global context to reconstruct the hyperspectral information at feature level. The experiment showed that our method produced the most accurate results with fewer artifacts.

Table 2 shows quantitative evaluation results of the competing methods in terms of RMSE in the [0–255] range, RMSERel, PSNR, and SSIM for the whole test set. This table shows an average per-pixel error drop of 42% in terms of RMSE and 46.6% in terms of RMSERel using SAPUNet-GAN compared to [3] over the test set. SAPUNet-GAN achieved comparable performance to [3]. SAPWNet-GAN yielded the best result in our experiment, with decreases of 0.82% and 5.7% compared with SAPUNet-GAN for RMSE and RMSERel, respectively.

**Figure 6:**
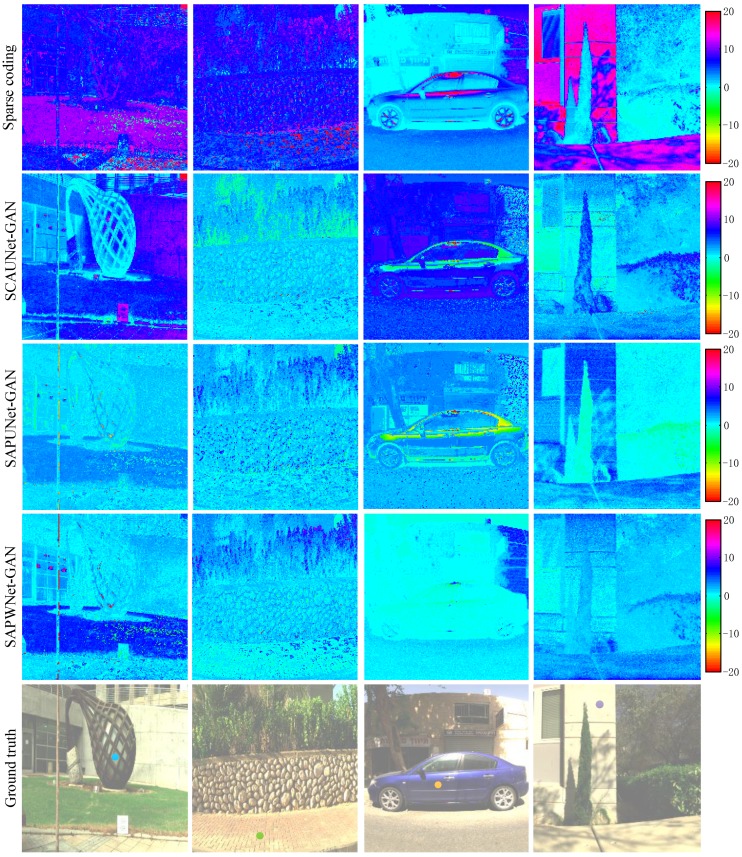
Visual comparison of the hyperspectral recovery of four selected images at 570 nm from the ICVL dataset [3]. From top to bottom: sparse coding [3], SCAUNet-GAN [30], SAPUNet-GAN, SAPWNet-GAN, and ground-truth.

**Figure 7:**
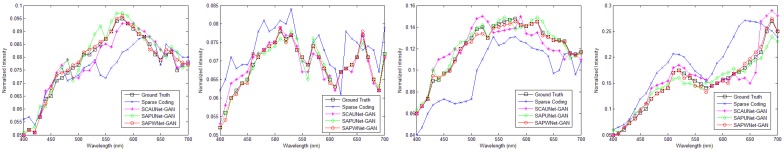
Spectral signatures of four selected spatial points identified by the colored dots from Figure 6 over 400–700 nm.

To evaluate the visual quality and spectral signature accuracy, Figure 6 shows four examples of error maps on a scale of ±255 from the ICVL dataset at a wavelength of 570 nm. SAPUNet-GAN and SAPWNet-GAN produced notably fewer errors than sparse coding and SCAUNet-GAN. For spectral signature accuracy evaluation, we conducted experiments using four spatial points of error maps over 400–700 nm as shown in Figure 7. We selected each spatial points identified by the colored dots in Figure 6. Compared with sparse coding, the results produced by GANs were much closer to the ground-truth. Our SAPUNet-GAN produced better results than SCAUNet-GAN. SAPWNet-GAN provide highest reconstruction fidelity compared with the selected alternatives.

### 4.2. Chakrabarti Dataset

We conducted an experiment with the model trained on the ICVL dataset. The results showed that the models provide generalization performance. Table 3 indicates that our methods outperformed the other algorithms for real-world images. Notably, the indoor performance of all methods was slightly worse compared to outdoors. The reason for this is the lack of luminance in indoor scenes and the generated models were trained on outdoor images. However, our methods still produced superior performance than the other state-of-the-art methods.

**Figure 8:**
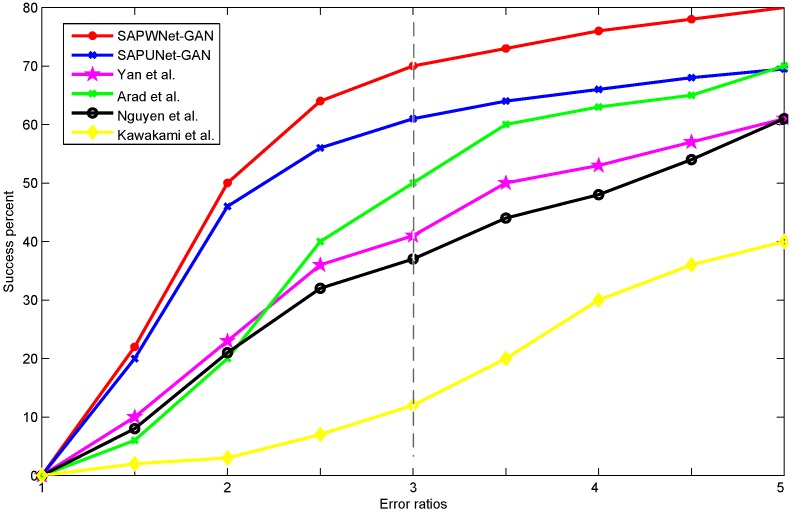
Quantitative evaluation on the Chakrabarti dataset in comparison with several state-of-the-art hyperspectral reconstructing methods: Yan et al. [16], Arad et al. [3], Nguyen et al. [14], Kawakami et al. [20].

Some complex scenes may result in larger absolute error even with a better visual quality compared to simple scenes. To normalize this effect, we calculated the success percentage of estimated pixels whose average absolute error was below specific values. We evaluated our method against the alternative algorithms in [3,6,14,16,20] using the proposed error metric. Arad et al. [3] created a sparse dictionary of spectra and corresponding RGB projections to map RGB vectors to spectra. Yan et al. [16] proposed a manifold-learning method to reconstruct hyperspectral images. Nguyen et al. [14] used a RBF network to learn the mapping from white-balanced RGB values from reflectance spectra. Parmar et al. [6] introduced controlled lighting to recover 31 spectral bands. Kawakami et al. [20] combined a low-resolution hyperspectral image and a high-resolution RGB image to acquire high-resolution hyperspectral data. Figure 8 shows the cumulative absolute error where higher curves indicate more accurate results. We measured the ratio between the absolute RMSE and the maximum radiation for each image on a scale of 0–255. The success percent indicates the percentage of test examples included in all spectral channels achieving an error ratio below the abscissa value. Empirically, the error ratio of 3 was considered the threshold of visually plausible results. Our method performed favorably against the alternatives.

### 4.3. Ablation Study for SAPWNet

We conducted an ablation experiment to demonstrate the effectiveness of the different components of the proposed SAPWNet with the ICVL dataset. We trained the different models with the same images as the training set, and used 40 new images as the test set for evaluation. We analyzed the following modules: feature pyramid, scale attention module, and W-Net, which implements boundary supervision in additional branch, as described above. These experiments showed that different factors affect the final result. As listed in Table 4, the hyperspectral reconstruction GAN based on U-Net (HSGAN) without any auxiliary components produces similar performance to Arad’s method [3]. We firstly implemented the feature pyramid structure, which produced 16.6%, 8.2%, and 6.1% improvements in terms of absolute RMSE, PSNR, and SSIM, respectively. When we replaced scale attention module with the feature pyramid to evaluate the performance, the improvements were almost 32.4%, 7.4%, and 12.1%, respectively. To optimize the visual quality of the recovered hyperspectral image, W-Net with boundary attention was introduced, which did not affect the learning of the main branch. Combining W-Net and the scale attention module produced the best result, creating 0.7%, 2.8%, and 1.7% improvements compared with no supervision network in terms of RMSE, PSNR and SSIM, respectively.

## 5. Conclusions

In this paper, we proposed two advanced adversarial CNN-based generative models for hyperspectral reconstructing from a single RGB image. We first designed SAPUNet, which establishes feature pyramids and uses an attention mechanism to select feature layers. The method uses local and global information corresponding to different sizes of receptive fields. Based on the promising SAPUNet results, we further presented the W-Net model, which replaces U-Net. This context fusion and boundary supervision at feature scales method yielded the best results. The experimental results showed that our approach both qualitatively and quantitatively outperforms the state-of-the-art methods. Nowadays, researchers are focusing on spectra reconstruction in the visible bands. Spectral recovery for infrared images deserves to be studied in the future. The sparsity of signals in the infrared range is probably lower than in visible bands. For this task, a larger number of input bands in the infrared range is required to achieve similar accuracy as with visible bands.

## Figures and Tables

**Table 1:** Model parameters of the discriminator.

#	Layer	Weight Dimension	Stride
1	Conv	64×32×5×5	2
2	Conv	64×64×5×5	1
3	Conv	128×64×5×5	2
4	Conv	128×128×5×5	1
5	Conv	256×128×5×5	2
6	Conv	256×256×5×5	1
7	Conv	512×256×5×5	2
8	Conv	512×512×3×3	4
9	Fc	512×1×1×1	0
10	Sigmoid	-	-

**Table 2:** A summary of the results of the conducted experiments including RMSE, RMSERel, PSNR and SSIM over the ICVL dataset [3].

Metric	Arad et al. [3]	Alvarez-Gila et al. [30]	SAPUNet-GAN	SAPWNet-GAN
RMSE	2.633	1.457	1.455	**1.445**
RMSERel	0.0756	0.0401	0.0398	**0.0378**
PSNR	27.641	-	31.647	**32.532**
SSIM	0.847	-	0.916	**0.932**

**Table 3:** The quantitative results on the dataset [40].

Datasets	Metric	Arad et al. [3]	Yan et al. [16]	SAPUNet-GAN	SAPWNet-GAN
Outdoor subset	RMSE	3.466	3.661	2.893	**2.687**
	PSNR	24.225	26.024	26.131	**26.316**
	SSIM	0.769	0.805	0.817	**0.835**
Indoor subset	RMSE	5.685	4.872	4.904	**4.782**
	PSNR	18.323	22.146	22.173	**22.468**
	SSIM	0.696	0.717	0.721	**0.757**

**Table 4:** Detailed analysis of the proposed SAPWNet with different settings. HSGAN, hyperspectral reconstruction GAN with U-Net generator; FP, feature pyramid without scale attention; SAM, scale attention module; W-Net, using W-Net replacing the U-Net.

Network	RMSE	PSNR	SSIM
HSGAN	2.586	27.224	0.756
HSGAN(FP)	2.155	29.463	0.817
HSGAN(SAM)	1.455	31.647	0.916
HSGAN(W-Net)	1.639	31.339	0.912
HSGAN(SAM+W-Net)	**1.445**	**32.532**	**0.932**
